# Open-Cell Rigid Polyurethane Foams from Peanut Shell-Derived Polyols Prepared under Different Post-Processing Conditions

**DOI:** 10.3390/polym11091392

**Published:** 2019-08-23

**Authors:** Guangyu Zhang, Yumin Wu, Weisheng Chen, Dezhi Han, Xiaoqi Lin, Gongchen Xu, Qinqin Zhang

**Affiliations:** 1 State Key Laboratory Base of Eco-chemical Engineering, College of Chemical Engineering, Qingdao University of Science and Technology, Qingdao 266042, China; 2 Shandong Provincial Key Laboratory of Biochemical Engineering, College of Marine Science and Biological Engineering, Qingdao University of Science and Technology, Qingdao 266042, China

**Keywords:** rigid polyurethane foams, bio-based polyols, peanut shell, floral foam

## Abstract

Bio-based polyurethane materials with abundant open-cells have wide applications because of their biodegradability for addressing the issue of environmental conservation. In this work, open-cell rigid polyurethane foams (RPUFs) were prepared with bio-based polyols (BBPs) derived from the liquefaction of peanut shells under different post-processing conditions. The influences of the neutralization procedure and filtering operation for BBPs on the foaming behaviors, density, dimensional stability, water absorption, swelling ratio, compressive strength, and microstructure of RPUFs were investigated intensively. The results revealed that a small amount of sulfuric acid in the polyols exhibited a great impact on physical and chemical properties of RPUFs while the filtering operation for those polyols had a slight effect on the above properties. The RPUFs prepared from neutralized BBPs possessed higher water absorption, preferable dimensional stability and compression strength than that fabricated from the non-neutralized BBPs. Moreover, the prepared RPUFs exhibited preferable water absorption of 636–777%, dimensional stability of <0.5%, compressive strength of >200 KPa, lower swelling rate of ca. 1%, as well as uniform cell structure with superior open-cell rate, implying potential applications in floral foam.

## 1. Introduction

Rigid polyurethane foams (RPUFs) are extensively used in numerous engineering applications, such as building and tank thermal insulation, structural support material, and composite wood due to their light weight, considerable specific strength, and superior heat insulation, etc. [1,2,3]. The major components for synthesizing RPUFs are isocyanate and polyols obtained basically from the petroleum industry. Due to the fast consumption of fossil oil reservoirs and environmental conservation, it is necessary to explore renewable feedstocks to substitute petroleum-based polyols for RPUFs production [4,5,6]. Biomass resources could make great contributions to the polyurethane industry development, because they are widely available, renewable and CO_2_-neutral feedstocks for the subsequent applications, especially in the preparation of RPUFs [7,8,9,10,11].

Generally, vegetable oils [4,8,12,13,14,15,16,17,18,19,20,21,22] and plant fibers [23,24,25,26,27,28,29] contain abundant hydroxyl groups or double bonds, which require chemical modification or liquefaction to generate bio-based polyols (BBPs) with proper hydroxyl numbers [10,11]. BBPs with hydroxyl numbers in the range of 200–550 mg KOH·g^−1^ would be suitable alternatives to replace the petroleum-based polyols for RPUFs synthesis [30]. As previously reported, foams prepared from BBPs could be used in thermal insulating materials with properties comparable to those of commercial products [8,11]. Agricultural residues such as crop straws and hulls, containing abundant polysaccharide and lignin with ample phenolic hydroxyl groups, are valuable biomass resources, which could be effectively converted into BBPs, as reported previously [31,32].

RPUFs are usually closed-cell foams due to the usage of low boiling point substance as foaming agents, resulting in the tightly reticular air barrier, low moisture vapor permeability and resistance to water. Therefore, closed-cell RPUFs have excellent thermal insulating properties and can be used for building thermal insulation materials [14,15,33]. However, in several new application areas, such as floral foam and noise reduction materials, RPUFs with high open-cells are required with properties of high water absorption [34] or sound absorption [35]. In the present studies, RPUFs with open-cell structure have rarely been reported. Typically, open-cell RPUFs can be synthesized by utilizing cell-opening agents, such as 1-butanol or the lithium salt of 12-hydroxystearic acid (Li-12HSA) [36].

This study was to synthesize the open-cell and bio-based RPUFs by using the liquefied products of peanut shell (defined as bio-based polyols, BBPs) as one of the dominant raw materials, where the BBPs were treated with four post-processing conditions. The effects of different post-processing conditions on the physical and mechanical properties, as well as the cell morphology of open-cell RPUFs have been intensively assessed.

## 2. Experimental

### 2.1. Materials

The liquefaction process of peanut shells for the preparation of BBPs could be found in previous report [31]. The properties of four BBPs are listed in Table 1, where A, B, C, and D stand, respectively, for the liquefied products of peanut shells filtered through a Buchner funnel with the filter paper (pore size: 30–50 μm) to remove residue (1.3 wt% relative to the original peanut shell) that cannot be liquefied by the solvents and neutralized with sodium hydroxide, the sample unfiltered and neutralized with sodium hydroxide, the sample filtered and non-neutralized, and the sample unfiltered and non-neutralized. Polymeric methylene-4,4′-diphenyl diisocyanate (PM-200) was obtained from Wanhua Chemical Group Co., Ltd. Triethylene diamine (A-33), stannous octoate (T-9) and silicone-based surfactant (L-580) were produced by Air Products & Chemicals, Inc. (Allentown, PA, USA).

### 2.2. Preparation of Open-Cell RPUFs

The open-cell RPUFs were synthesized through a one-step method. The content of all the additives was a relative mass ratio to the BBPs. Firstly, the BBPs (100 wt %), blowing agent (distilled water, 2 wt%), L-580 (2–3 wt %) and complex catalysts (A-33 of 0.75–1.00 wt % and T-9 of 0.3–0.4 wt %) were fully blended in a 500 mL plastic beaker with stirring (800 rpm) for one minute. Then the pre-weighted PM-200 (where NCO index was 1.00–1.05 and the isocyanate content was calculated in our previous study [37]) was poured into the beaker rapidly under continuous stirring of another 90–120 s. Finally, the homogeneous mixture rose freely and then was cured at room temperature for 24 h before taking it out of the plastic beaker. The samples were kept at ambient temperature for at least three days before their properties were measured. The RPUFs from BBPs A, B, C and D were defined respectively as RPUF-A, RPUF-B, RPUF-C and RPUF-D.

### 2.3. Characterization and Property Testing of RPUFs

The gel time and free rise time of RPUFs were tested according to the standard “cup-test” in ASTM D7487-13E1 using a digital timer. Each test was conducted repeatedly at least five times for minimizing experimental error. The inner temperature of RPUF was measured by inserting the thermometer into the mixture during the foaming process to record the maximum value of the temperature. The density of RPUF was measured according to GBT 6343-2009. Prior to the test, the samples with the size of 50 mm × 50 mm × 50 mm were kept at the temperature of 25 °C and relative humidity of 50% for at least 16 h. Dimensional stability of RPUFs was measured in accordance with GBT 8811-2008 over the foams with the size of 100 mm × 100 mm × 25 mm as the temperature was −25 °C and 85 °C, respectively. The compressive strength test of RPUFs (50 mm × 50 mm× 50 mm) was carried out according to GB T 8813–2008 using an electronic universal testing machine (H10KS, Hounsfield, England) under the loading speed of 5 mm·min^−1^. The water absorption and swelling ratio in the water of RPUFs (150 mm × 150 mm × 50 mm) were tested based on method A and method B in GBT 8810–2005 under the temperature of 25 °C and relative humidity of 50%. The porosity and cell microstructure of RPUFs were observed using a cold-field emission scanning electron microscope (S-4800, Hitachi) with the cross-section sampling to the foam growth direction after coating with gold.

## 3. Results and Discussion

### 3.1. Foaming Behaviors

**Figure 1:**
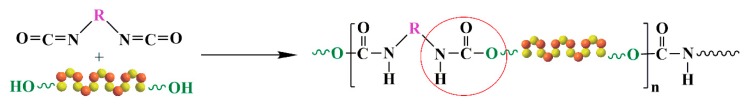
Gelling reaction between the isocyanate and polyols.

The reactions of isocyanate with water and polyols are intense exothermic processes. The carbon dioxide generated from the blowing reaction between the isocyanate and water would act as foaming gas to expand bubbles. Meanwhile, the backbone of the urethane group is formed from the gelling reaction between isocyanate and polyols with different molecular weight as shown in Figure 1.

**Figure 2:**
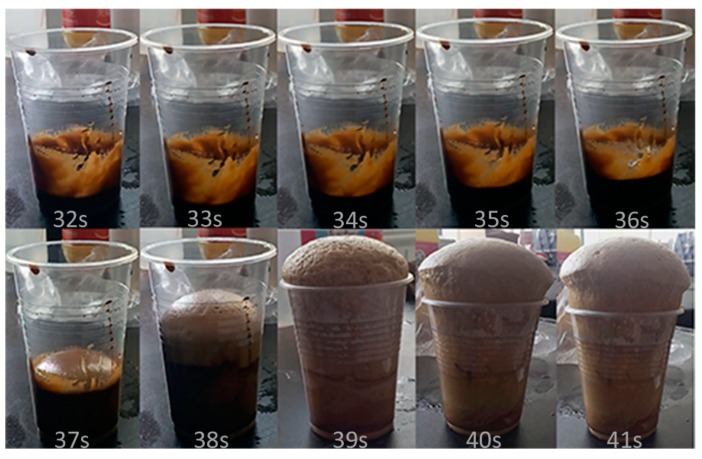
Foaming process of RPUF-B prepared from unfiltered bio-based polyols (BBPs) with neutralization by sodium hydroxide.

**Figure 3:**
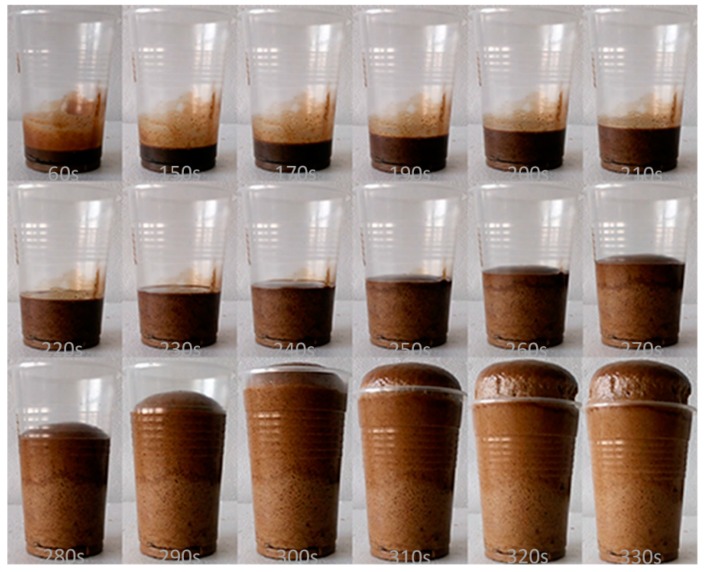
Foaming process of RPUF-D prepared from unfiltered BBPs without neutralization by sodium hydroxide.

The gel time and the free rise time were recorded during the foaming process and listed in Table 2. It can be found that the gel time and free rise time of RPUF-A and -B could be dramatically reduced in comparison with that of the RPUF-C and -D due to the use of the BBPs neutralized with sodium hydroxide, indicating the great influence of neutralization procedure of BBPs on the synthesis of the RPUFs. For instance, using the filtered BBPs, the gel time and free rise time significantly increased from 24 and 39 s for RPUF-A to 449 and 578 s for RPUF-C, respectively. Moreover, the filtration process of BBPs had a slight effect on both the gel time and free rise time of foams, illustrating that the preparation of BBPs without filtration process could save the time as well as the cost of the final RPUFs. The foaming variation with the elevated free rise time for RPUF-B (Figure 2) and RPUF-D (Figure 3) further clearly verified that the free rise time of foam prepared from neutralized polyols was substantially shortened. The alkaline amine catalyst A-33 could fully exhibit its catalytic performance for promoting the reaction of isocyanate and water to generate carbon dioxide during the foaming process due to the removal of sulfuric acid through the neutralization procedure.

**Figure 4:**
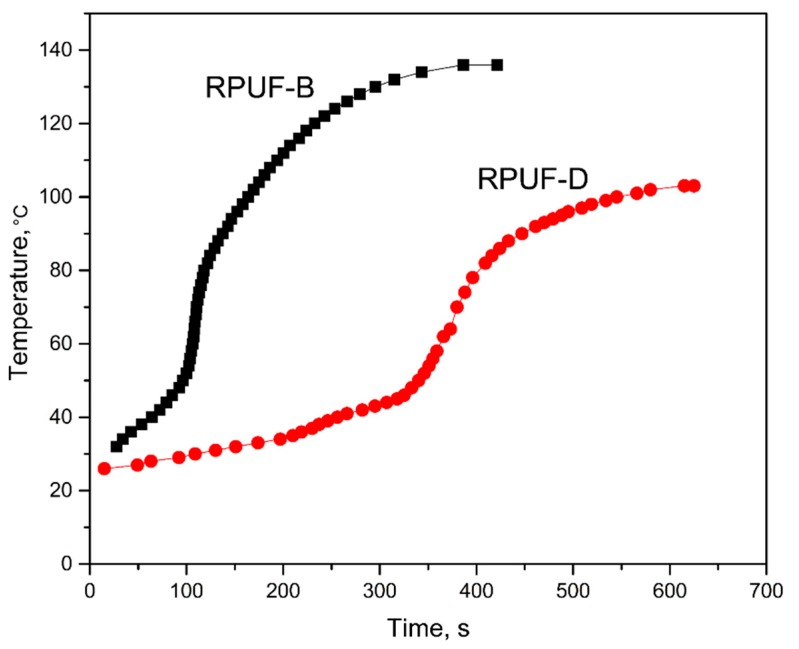
Inner temperature variation of foams prepared from unfiltered BBPs with (RPUF-B) and without (RPUF-D) neutralization by sodium hydroxide.

**Figure 5:**
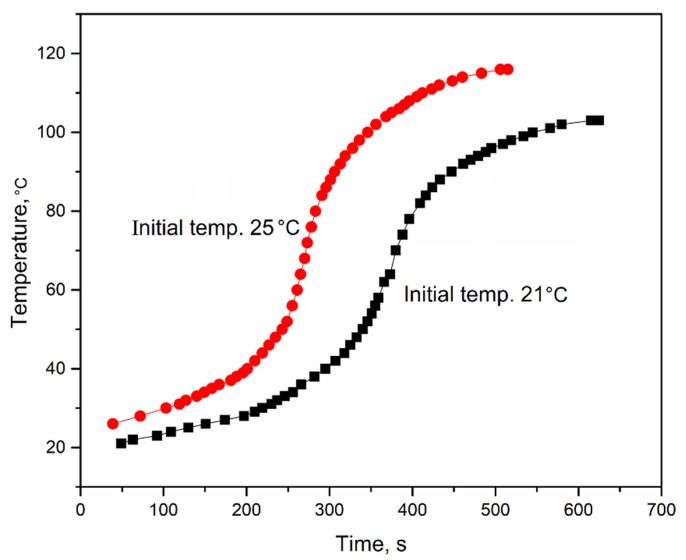
Inner temperature variation during the foaming process under different initial temperatures of the foaming mixture.

Figure 4 shows the inner temperature variation trend of RPUF-B and RPUF-D during the foaming process. It could be seen that the inner temperature of the two samples increased with respect to the test time. The inner temperature of RPUF-B reached the maximum value of 136 °C after 386 s, which is relatively higher and faster than that (103 °C after 625 s) of RPUF-D. The presence of sulfuric acid in the BBPs without neutralization would react with alkaline amine catalyst A-33 to slow down the reaction between the isocyanate and water during the preparation of RPUF-D, resulting in the mild exothermic process, thus the relatively low inner temperature. This is consistent with the observation of Figure 2 and Figure 3. Therefore, the neutralization procedure could be necessary to prepare the BBPs for the subsequent RPUFs synthesis. It was also found that the initial temperature of foaming mixture had an obvious effect on the inner temperature during the foaming process (Figure 5), illuminating that the high initial temperature of foaming mixture can accelerate the reaction rate to shorten the overall reaction time.

### 3.2. Apparent Density

**Figure 6:**
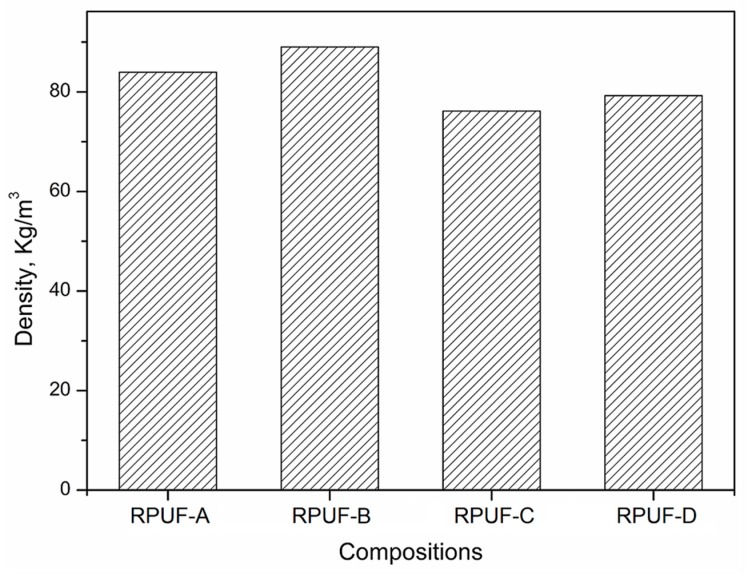
The apparent density of prepared RPUFs.

The apparent density of RPUFs is presented in Figure 6. It can be found that the apparent density of all RPUFs was in the range of 75–90 Kg·m^−3^, suggesting the formation of the dense structure. Furthermore, the apparent density of RPUF-A and B was higher than that of RPUF-C and D, respectively. The relatively high inner temperature of the RPUF-A and B would facilitate the formation of the framework of the urethane group in the stage of the gel reaction between the BBPs and isocyanate, resulting in the high apparent density of the prepared RPUFs. Thus, the remaining sulfuric acid in the BBPs exhibited a certain impact on the properties of the final RPUFs and should be removed by the neutralization with sodium hydroxide.

### 3.3. Dimensional Stability and Water Absorption

The dimensional stability of RPUFs under different temperature is listed in Table 3. As expected, the dimensional changes of RPUFs under low temperature and thermal treatment were unregulated and negligible (−0.07% to 0.50%), indicating that the prepared RPUFs is favorable for the practical engineering application in the wide range of temperature.

Water absorption is usually associated with the open-cell ratio and density. As shown in previous work on the RPUF from rapeseed oil polyol with a high content of closed cells, the water absorption foams are less than 10% [16]. However, as listed in Table 4, the four prepared RPUFs in this study possessed substantially higher water absorption (636%–777%) as well as the extremely low swelling ratios (around 1%), implying the high open-cell ratio and density of prepared RPUFs. The RPUFs with the properties of high water absorption, low swelling ratio, and suitable density are favorable for the application of floral foam [38].

### 3.4. Mechanical Properties

**Figure 7:**
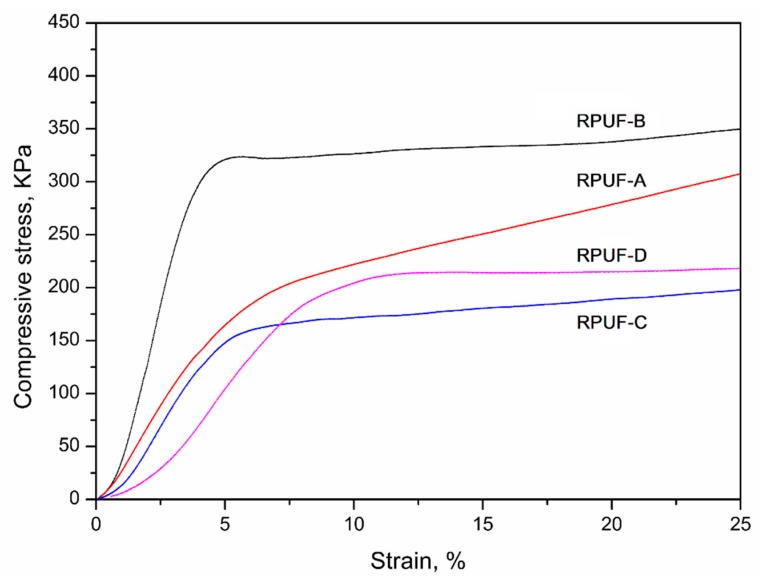
Stress-strain curves of the prepared RPUFs for the determination of the compressive strength.

The mechanical properties of prepared RPUFs were evaluated by compressive strength test and the results are illustrated in Figure 7 and Table 5. The compressive strength of foams prepared from the neutralized BBPs (RPUF-A and B) was substantially higher than that of RPUF-C and D, indicating that the neutralization process of the BBPs would significantly influence the compressive strength of the subsequent RPUFs. Moreover, the unfiltered BBPs containing few residues can strengthen the mechanical strength of the RPUFs, resulting in the higher compressive strength of the foams derived from the unfiltered BBPs in comparison with foams from filtered BBPs. Typically, the compressive strength of RPUF-B was obviously higher than that of RPUF-A. The mechanical test results are also in accordance with the density results (Figure 6); that is, the foam with high density also exhibited the superior mechanical strength. Except for density, the compressive strength of RPUFs is also relative to the cell size and shape of the final foams. The RPUFs with regular cell shape and uniform cell size usually possessed high compressive strength [34,39]. This can be proved by the morphology investigation in the following discussion. Furthermore, the compressive strength of prepared RPUFs in this work is superior in comparison with the foams from others’ work [16,26].

### 3.5. Cell Morphology

**Figure 8:**
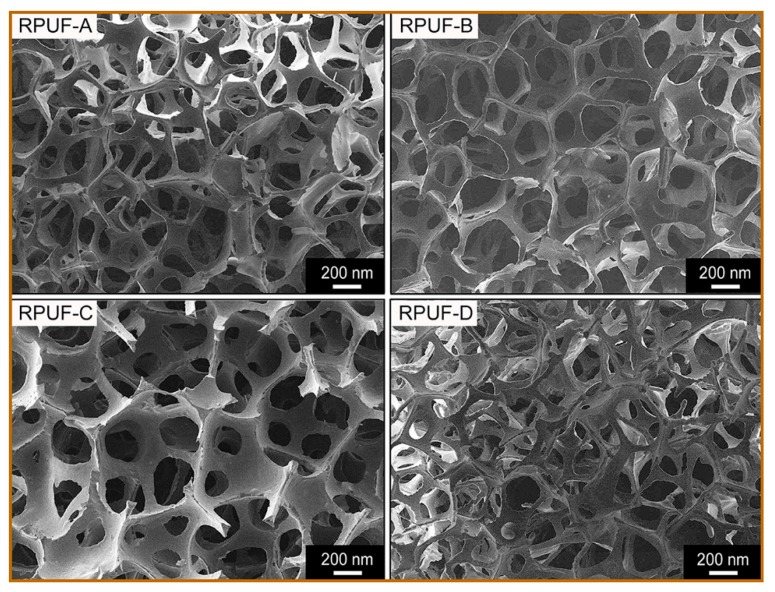
SEM images of the prepared RPUFs.

SEM images (Figure 8) of prepared RPUFs reveal that the foam cells have a regular shape and uniform size, indicating the isotropic growth of the bubble during the foaming process. This result also verified the conclusion from the analysis of the compressive strength test. Furthermore, the cells are approximately hexagonal and completely opened. In the foaming process, the slow-gelling reaction rate allowed the bubbles to easily escape from the matrix before it forms the firm struts. Finally, an equilibrium was reached between the gelation and blowing reaction, leading to the formation of RPUFs with uniform cell size. The superior performance of the prepared RPUFs enable them to be potentially used as floral foam.

## 4. Conclusions

The bio-based rigid polyurethane foams (RPUFs) with high open-cell ratio were successfully prepared with bio-based polyols (BBPs) derived from the liquefaction of peanut shells under different post-processing conditions. Compared to the filtration post-processing of BBPs, the neutralization of BBPs with sodium hydroxide would significantly influence the properties of the final foams due to the elimination of the small amount of sulfuric acid, which could slow down the reaction between the isocyanate and water during the preparation of RPUFs. The RPUFs prepared from neutralized BBPs exhibited the suitable density, superior compressive strength, especially high water absorption of 636%–777% and low swelling ratio of ca. 1% as well as uniform cell structure with high open-cell rate. These properties of the obtained RPUFs are favorable for application as a floral foam.

## Figures and Tables

**Table 1:** Properties of bio-based polyols.

Sample	OH Number, mgKOH·g^−1^	Acid Number, mgKOH·g^−1^	Viscosity(25°C), mPa·s	Color
A	451.9	1.0	47	black
B	473.3	1.0	143	black
C	451.9	8.9	47	black
D	473.3	8.9	143	black

**Table 2:** The gel time and free rise time of the foaming process in different reaction conditions.

Samples	Gel Time, s	Free Rise Time, s
RPUF-A	24	39
RPUF-B	28	41
RPUF-C	449	578
RPUF-D	480	593

**Table 3:** Dimensional stability of RPUFs at different temperature.

Samples	−25 °C			85 °C		
	Length, %	Width, %	Height, %	Length, %	Width, %	Height, %
RPUF-A	−0.13	−0.07	−0.03	0.02	0.08	0.07
RPUF-B	−0.12	−0.05	−0.16	0.13	0.26	0.18
RPUF-C	−0.35	−0.07	−0.15	0.07	0.50	0.15
RPUF-D	−0.34	−0.10	−0.27	0.06	0.27	0.20

**Table 4:** Water absorption and swelling ratio of RPUF in different compositions.

Samples	Water Absorption, %	Swelling Ratio, %
RPUF-A	687	1.06
RPUF-B	777	1.05
RPUF-C	636	1.09
RPUF-D	678	1.03

**Table 5:** Mechanical properties of RPUFs.

Samples	Maximum Pressure, N	Compression Strength, KPa	Stress-Strain		
			10%, KPa	20%, KPa	25%, KPa
RPUF-A	63.4	248.5	221.9	278.6	307.5
RPUF-B	89.5	350.7	326.4	337.7	349.6
RPUF-C	51.0	200.1	171.4	189.0	197.9
RPUF-D	59.2	231.7	204.4	215.1	218.3
